# Physicochemical Compatibility and Stability of Linezolid with Parenteral Nutrition

**DOI:** 10.3390/molecules24071242

**Published:** 2019-03-29

**Authors:** Szymon Tomczak, Maciej Stawny, Katarzyna Dettlaff, Maria Kieliszek, Daria Słomińska, Anna Jelińska

**Affiliations:** Department of Pharmaceutical Chemistry, Poznan University of Medical Sciences, 6 Grunwaldzka, 60-780 Poznań, Poland; mstawny@ump.edu.pl (M.S.); dettlaff@ump.edu.pl (K.D.); mary.kieliszek@gmail.com (M.K.); slominska.daria@gmail.com (D.S.); ajelinsk@ump.edu.pl (A.J.)

**Keywords:** drug design/delivery, total parenteral nutrition mixtures, linezolid, compatibility, stability

## Abstract

Patients referred to intensive care units (ICU) require special care due to their life-threatening condition, diseases and, frequently, malnutrition. Critically ill patients manifest a range of typical physiological changes caused by predominantly catabolic reactions in the body. It is necessary to provide the patients with proper nutrition, for example by administering total parenteral nutrition (TPN). The addition of linezolid to TPN mixtures for patients treated for linezolid-sensitive infections may reduce the extent of vascular access handling, resulting in a diminished risk of unwanted catheter-related infections. The compatibility and stability studies were conducted of linezolid in parenteral nutrition mixtures of basic, high- and low-electrolytic, high- and low-energetic and immunomodulatory composition. Mixtures containing linezolid were stored at 4–6 °C and 25 °C with light protection and at 25 °C without light protection for 168 h. In order to evaluate changes in the concentration of linezolid a previously validated reversed-phase HPLC method with UV detection was used. It was found that linezolid was stable at 4–6 °C in the whole course of the study whereas at 25 °C it proved stable over a period of 24 h required for administration of parenteral nutrition mixtures. The TPN mixtures demonstrated compatibility with linezolid and suitable stability, which were not affected by time or storage conditions.

## 1. Introduction

Patients referred to intensive care units (ICU) require special care due to their life-threatening condition, diseases and, frequently, malnutrition. Critically ill patients manifest a range of typical physiological changes caused by predominantly catabolic reactions in the body [1]. This results from the secretion of hormones (e.g., glucagon, cortisol), catecholamines and proinflammatory cytokines as well as the increased resistance of tissues to anabolic hormones (e.g., insulin and insulin-like growth factor). As a consequence, proteolysis and muscle loss (up to 1 kg per day) together with lipolysis and body mass reduction are observed [2,3,4,5], leading to a greater death rate [6]. For these reasons it is necessary to provide the patient with proper nutrition, alongside the management of the target health problem [7], for example by administering total parenteral nutrition (TPN). According to the 7th recommendation of the American Society for Parenteral and Enteral Nutrition (ASPEN), TPN is to be applied when the patient is unable to eat [2].

The knowledge of nutritional needs, the availability of soluble pharmaceutical forms allowing intravenous administration and the provision of safe access to the veins have ensured the safety and effectiveness of parenteral nutrition. A vital consideration regarding effective parenteral nutrition is the need to provide patients with a stable nutritional mixture composed according to their therapeutic requirements [8,9].

Total parenteral nutrition mixture (TPN) is a multicomponent drug consisting of over 50 ingredients mixed within one container, which involves some risk of interaction between the components, auxiliary substances and packaging [10,11]. TPN is expected to be physicochemically and microbiologically stable in addition to meeting all requirements for parenteral drugs during preparation, storage and administration [11,12,13]. Other important considerations include defining the shelf-life of TPN and the possibility of adding drugs (e.g., vitamins and trace elements), whose stability may be affected by the presence and concentrations of TPN components (amino acids, glucose, lipid and electrolytes) as well as storage conditions, e.g., temperature and light exposure [13,14].

It has been reported that the stability of TPN mixtures is affected to the greatest extent by storage time and temperature as well as exposure to light, although the mixture content (e.g., amino acid preparation, lipid emulsion, electrolyte concentration and vitamins) may also influence TPN mixture stability [13,14,15,16].

The greatest risk facing critically ill patients is posed by infections caused particularly by drug-resistant bacteria. It has been reported that linezolid is one of the drugs effective in the treatment of this kind of infections [17,18,19,20,21].

**Figure 1:**
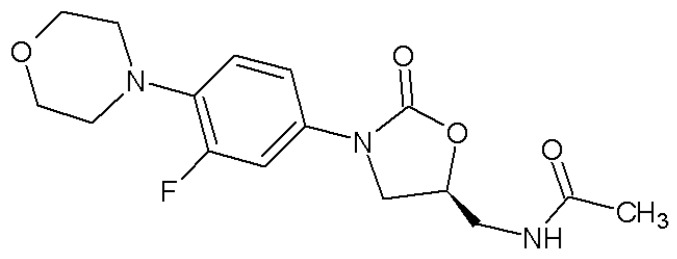
Chemical structure of linezolid.

Linezolid (Figure 1) is a synthetic, antibacterial agent that belongs to a new class of antimicrobials, the oxazolidinones [22,23,24]. It is used exclusively in hospital treatment. Linezolid selectively inhibits bacterial protein synthesis via a unique mechanism of action. [25,26,27]. The activity spectrum of linezolid comprises mainly aerobic Gram-positive bacteria, including vancomycin resistant enterococci (VRE), methicillin-resistant *Staphylococcus aureus* (MRSA) and penicillin-resistant pneumococci [22,24,27].

Regarding the determination of linezolid, the following methods have been reported: high-performance liquid chromatography (HPLC) [28,29,30,31], high-performance thin-layer chromatography (HPTLC) [32,33], and capillary electrophoresis [34].

Forced degradation studies were performed on linezolid in solutions—sodium chloride 0.9%, sodium lactate, glucose 5% and glucose 10%—containing 2.0 mg/mL linezolid. The solutions were stored at 25.0 ± 0.1 °C for 34 days. The effect of temperature on the stability of linezolid was investigated in 0.1 M sodium hydroxide. At 70 °C the observed rate constants were determined, which allowed establishing the pH rate profile. A stability study of IV linezolid in the preparation ZYVOX was performed at 70 °C for 72 h. It was found that linezolid demonstrated adequate stability for the preparation of intravenous fluids for clinical administration. By using LC and LC-MS/MS systems it was possible to determine the stability of linezolid and its six impurities [35].

The application of linezolid is recommended in infections with vancomycin resistant *Enterococcus faecium*, hospital-acquired pneumonia caused by methicillin-sensitive *Staphylococcus aureus* (MSSA), methicillin-resistant *Staphylococcus aureus* (MRSA) or *S. pneumonia*, including resistant strains, complicated skin infections, diabetic foot infections without attendant bone marrow infections caused by MSSA or MRSA, *S. pyogenes* and *S. agalactiae*, non-complicated skin infections caused by MSSA or *S. pyogenes*, non-hospital-acquired pneumonia (MSSA and *S. pneumonia*) including multi drug resistant (MDR) strains, infections of bones and joints including post-prosthetic-implantation infections, enterococci-induced endocardium infections and pneumococci-induced meningitis [23,24,36]. Linezolid is a time-dependent antimicrobial agent with a reduced post-antibiotic effect. The most suitable pharmacokinetic/pharmacodynamic (PK/PD) parameters defining its activity are time with serum concentrations higher than the minimum inhibitory concentration (*T* > MIC) during the dosing interval [37].

The intravenous administration of linezolid improves PK/PD parameters in critically ill patients [37] and it is indicated in malnutrition [38]. Therefore, the addition of linezolid to TPN may improve the effectiveness of therapies for critically ill patients on condition that stability and compatibility studies prove the combination of linezolid and TPN to be therapeutically safe.

The present study aimed to determine the physicochemical stability and compatibility of linezolid in TPN mixtures. Stability studies were performed at 4.0 °C without light exposure and at 25 °C with and without light exposure. Changes in the concentrations of linezolid were determined by using an HPLC method and compatibility was evaluated by visual inspection and the determination of lipid emulsion particle size, zeta potential and pH. The methodology was also graphical presented (Supplementary Material Scheme S1). 

## 2. Results and Discussion

The determination of linezolid in TPN mixtures was performed by using an HPLC method. It was found to be selective, linear in the drug concentration ranges of the study and precise, and as such suitable for linezolid determination in 3-in-1 TPN mixtures after *n*-hexane extraction. The TPN mixtures, prepared according to the compositions shown in Table 1, were white, odorless and homogenous. All of the compositions are widely used in hospital pharmacy.

### 2.1. Linezolid Determination in TPN Mixtures

**Figure 2:**
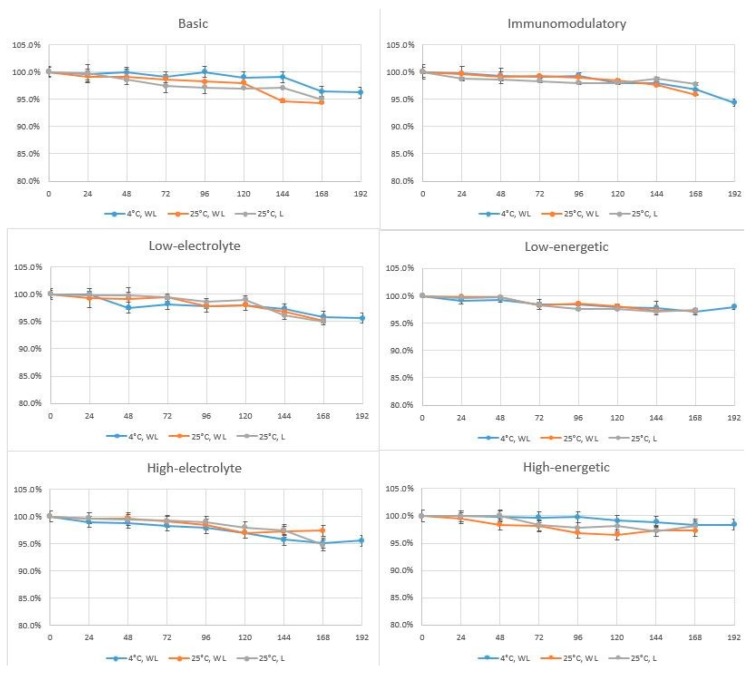
Percentage changes of linezolid concentration in TPN mixtures during experiments. WL—without light, L—with light.

The changes of linezolid concentration are shown in Figure 2. The linezolid peak area after addition to the TPN mixtures (t = 0 h) was taken as 100% of the nominal value to which the linezolid peak areas observed over the next days of the study were compared.

Linezolid demonstrated the required stability in TPN mixtures during the entire study period, irrespective of storage conditions and mixture type. It appears that linezolid may be added to TPN mixtures and administered in this form as infusion over a period of 16–24 h. However, pharmacokinetic studies are necessary to confirm that assumption. 

### 2.2. Compatibility Studies

The compatibility studies of linezolid in TPN mixtures aimed in the course of the whole study to visually examine the samples for such changes as discoloration, creaming and stratification, and to measure mixture pH, particle size and zeta potential. Visual inspection did not indicate any changes in the TPN mixtures with and without linezolid.

**Figure 3:**
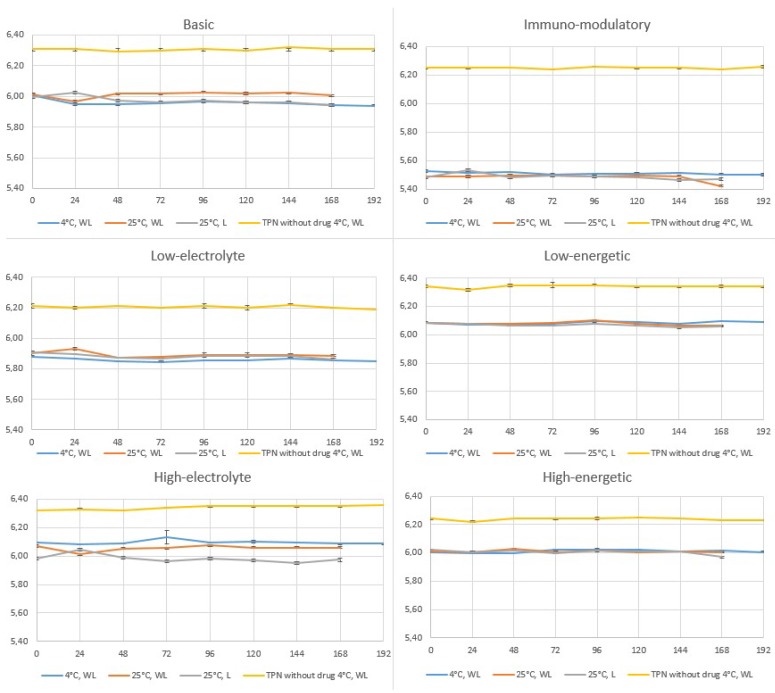
pH value changes of TPN mixtures with linezolid during storage time (WL—without light, L—with light.).

pH measurements of TPN mixtures with and without linezolid showed only slight changes over a 7-day period (±0.01 to ±0.09) (Figure 3). TPN mixtures of different compositions stored at 4–6 °C demonstrated varying pH, with the greatest changes observed for immunomodulatory and high-electrolyte compositions (Figure 3). Considerably different pH values were shown by the immunomodulatory composition, containing lipid emulsion rich in fish oil. The use of different electrolytes in varying proportions also affected the pH of the low- and high-electrolyte mixtures. The addition of a 1200 mg daily dose of linezolid resulted in a decrease in the pH of all TPN mixtures. Although a pH decrease to less than 5.5, which occurred in the immunomodulatory TPN mixture, may destabilize the oil-water system [39], no such destabilization was observed during the study.

Other parameters determining the stability of TPN mixtures is the polydispersity index (PDI) and the size of lipid emulsion particles. The PDI expressing the non-homogeneity of the particles in a given system, is measured together with the particle size and should range from 0.05 to 0.7 [40]. In the present work, the PDI was in that range, which proved the suitability of the measurement technique used and confirmed that the DLS method can be applied for measuring the size of lipid emulsion particles in the TPN mixtures examined in the study.

Since TPN mixtures are administered parenterally, lipid emulsion particle size is one of the key parameters affecting therapeutic safety. The largest endogenous lipoproteins and chylomicrons are known to be 75–600 nm in diameter. In the case of lipid emulsions for parenteral administration, lipid emulsion particle size should not exceed 500 nm to prevent blockage of capillary vessels leading to serious clinical complications such as damage to pulmonary, hepatic and/or retinal blood vessels by larger particles [39,41]. The mean lipid emulsion particle size measured by the DLS method must not exceed 500 nm, according to US Pharmacopeia [42].

**Figure 4:**
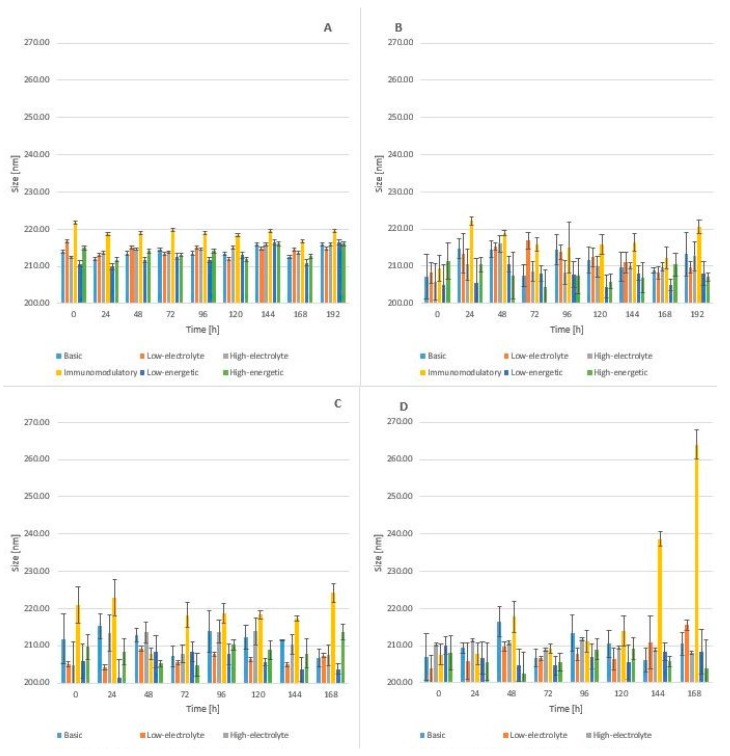
The mean particle size for (**A**) Mixtures without linezolid (**B)** With linezolid storage in 4–6 °C without light (**C**) With linezolid in 25 °C without light (**D**) With linezolid 25 °C with light.

The mean particle size for mixtures without linezolid ranged from 211.0 nm to 216.70 nm after preparation and during storage from 210.70 nm to 221.70 nm at day 7 (Figure 4). Upon the addition of a daily dose of linezolid, the mean particle size decreased to 204.97 nm for low-energetic composition. At a 7th day storage temperature of 25 °C, the mean particle size ranged from 203.70 nm to 238.63 nm for all mixtures without immunomodulatory because in this composition the parameter reached 264.00 nm and was bigger than the rest.

Mixtures for parenteral nutrition, as oil-water emulsions, are dynamic systems in which the particles move continuously according to Brownian motion principles. Therefore, slight changes in the lipid emulsion particle size are commonly observed.

The fact of the diminishing mean particle size occurring in all types of TPN mixtures after the addition of linezolid and the observation of a slight increase in this parameter during storage, with the exception of immunomodulatory composition, indicated that the presence of linezolid did not significantly affect the stability of the TPN mixtures studied.

It is particularly important that in all types of TPN mixtures one particle fraction was observed in the course of the whole study.

**Figure 5:**
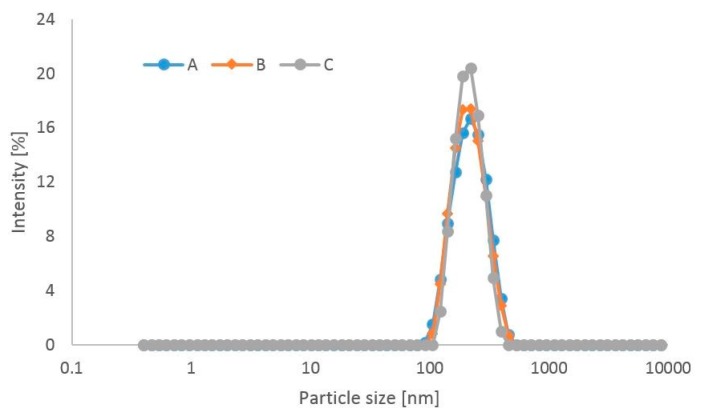
Particle size distribution in the TPN mixture of basic composition (**A**) with linezolid after preparation (**B**) with linezolid at day 7 of the study stored at 4 °C and protected from light (**C**) without linezolid after preparation.

Figure 5 shows particle size distribution in the TPN mixture of basic composition with linezolid after preparation and at day 7 of the study stored at 4–6 °C and protected from light. All TPN mixtures studied were found to contain one particle fraction.

**Figure 6:**
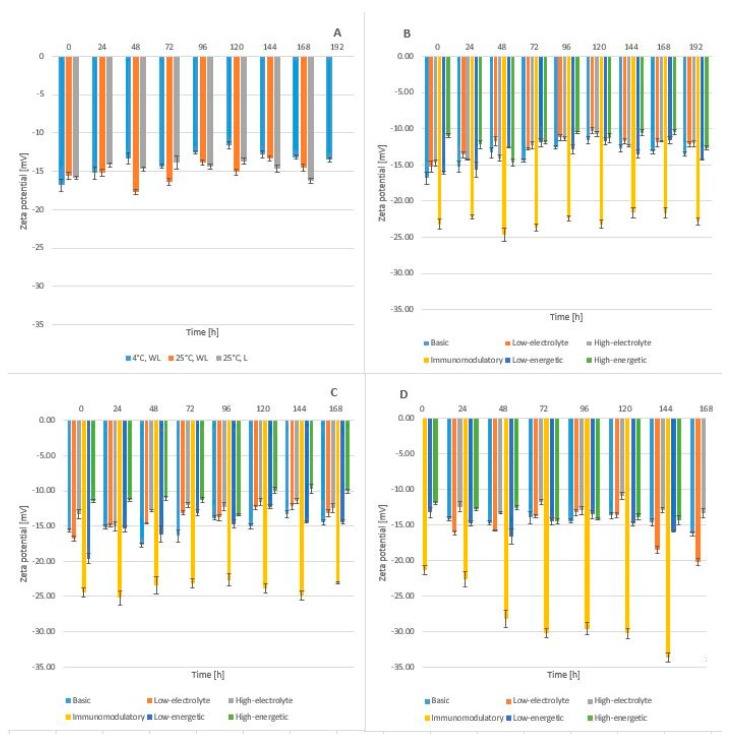
The zeta potential for mixtures (**A**) Without linezolid (**B**) With linezolid storage in 4–6 °C without light (WL) (**C**) 25 °C without light (**D**) 25 °C with light (L).

Zeta potential defines the charge on the surface of lipid emulsion particles and allows assessment of electrostatic interaction between the particles. The zeta potential of lipid emulsions containing phospholipids ranges from −40 mV to −50 mV [43]. It can be reduced by adding amino acids and ions of mono- (potassium, sodium) and bivalent (magnesium, calcium) [43,44,45,46]. For the TPN mixtures without linezolid, the highest zeta potential values were found for high-electrolytic composition whereas the lowest for immunomodulatory composition (Figure 6).

The addition of linezolid to the TPN mixtures decreased zeta potential values, thus stabilizing the lipid emulsions. A marked decrease in zeta potential was observed for the immunomodulatory TPN mixture. This may result from the presence of another lipid emulsion containing 50% medium-chain (MCT), 40% long-chain (LCT) and 10% omega-3-triglycerides. It may be assumed that the oil-water system is stabilized by the addition of omega-3 acids leading to an increase in absolute values of zeta potential. For the TPN mixtures stored at 25 °C, a tendency for a decrease in zeta potential values was observed especially in those mixtures that were not protected from light.

Linezolid showed required stability in the TPN mixtures. Its content at 168 h of storage differed only slightly from the initial value. This indicates that the stability and concentration of linezolid in a therapeutic dose do not fall below the required limits during infusion. It is necessary to establish whether linezolid may be added to TPN mixtures while ensuring therapeutic safety in order to apply such a treatment option in the case of infections requiring linezolid as an antidote in patients dependent on parenteral nutrition. However, catheter-related bloodstream infections (CRBSI) may not be treated by administering the above-mentioned modality because different regimens are indicated in such cases.

It was shown that the presence of linezolid in the TPN mixtures examined in this work under different thermal and light conditions did not cause any interactions. Although the study demonstrated its stability and compatibility with the TPN mixtures, for reasons of therapeutic safety and effectiveness, the addition of linezolid to TPN mixtures requires further pharmacokinetic studies.

## 3. Materials and Methods

### 3.1. Materials

Linezolid raw material for HPLC (LOT:08A2F60JPU9, AK Scientific, Union City, CA, USA), Linezolid Polpharma (Polpharma S.A., Starogard Gdański, Poland) 300 mL infusion bags containing 600 mg of linezolid and auxiliary substances such as glucose monohydrate, sodium citrate, citric acid, hydrochloric acid (0.1 M), sodium hydroxide (0.1 M) and water for injection were used. The content of sodium ions and glucose in 1 mL of the preparation was 0.37 mg (0.113 g in a dose) and 45.67 mg (13.702 g in a dose), respectively. The pH ranged from 4.3 to 5.3. Solutions used to TPN preparation Aminoplasmal^®^ B. Braun 10% E, Aminoplasmal HEPA^®^ B. Braun, 40% Glucose B., Lipofundin^®^ MCT/LCT 20, LIPIDem^®^, Aqua pro iniectione, Natrium Chloratum 10% (all purchased from B. Braun Melsungen AG, Melsungen, Germany), Kalium Chloratum 15% WZF (WZF Polfa S.A., Warsaw, Poland), Calcium gluconate 10% (Added Pharma, Oss, The Netherlands), Glycophos (Fresenius Kabi AB Uppsala, Sweden), Inj. Magnesii sulfurici 20% (Polpharma S.A., Starogard Gdański, Poland).

### 3.2. Reagents

*n*-Hexane (VWR-Chemicals, Fontenay-sous-Bois cedex, France). Other chemicals and solvents (Merck, Darmstadt, Germany). High quality pure water (Exil SA 67120, Millipore, Molsheim, France).

### 3.3. Preparation of Total Parenteral Nutrition Mixtures

The study involved the use of a parenteral nutrition mixture with a composition designed to meet the energetic needs of a 70 kg adult male on parenteral nutrition due to the short bowel syndrome, engaged in moderate physical activity [47]. TPN mixtures were prepared under aseptic conditions in a clean room under a laminar airflow, using a Pinnacle automatic compounder (B. Braun), coupled to the software TPN Manager^®^ (B. Braun Medical Inc., Ave Bethlehem, PA, USA). Six parenteral nutritional mixtures were prepared differing in the content of glucose, electrolytes and amino acids and in the type of lipid emulsions, of which one contained unsaturated omega-3 fatty acids (Lipidem^®^, B. Braun Melsungen AG, Melsungen, Germany) with immunomodulatory activity (Table 1). Following the ASPEN recommendations [48], visual inspection of all TPN mixtures was performed to detect color changes, creaming or phase separation.

### 3.4. Preparation of TPN Mixtures with Linezolid

To prepare TPN mixtures with linezolid (a daily linezolid dose of 1200 mg), 600 mL of the preparation was diluted to 2500 mL with the TPN mixtures in ethylene-vinyl acetate (EVA) bags to reach 0.48 mg/mL of linezolid. To avoid overhydration and an excess of sodium ions, the TPN mixtures were adjusted by decreasing the amounts of water, glucose and sodium ions contained in the infusion fluids. Each sample was prepared in triplicate. The 7-day test started with the addition of linezolid to the mixtures (t = 0 h), which were next stored at 25 ± 1 °C (with and without light protection) and at 4–6 °C with light protection. The samples stored at 4–6 °C were next kept for 24 h at room temperature with light exposure to simulate infusion (192 h). A daily visual inspection was conducted to detect emulsion stratification, sedimentation or creaming. The stability and compatibility studies comprised an analysis of the effect of temperature and light on the stability of linezolid in TPN mixtures and on the stability of TPN mixtures after the addition of linezolid.

### 3.5. Extraction of Linezolid from TPN Mixtures for HPLC Analysis

3.0 mL of the so-obtained mixtures with linezolid were introduced into 10 mL plastic vials and 5.0 mL of n-hexane were added. The vials were agitated for 15 min (MPW Med. Instruments, Warsaw, Poland) and centrifuged (GFL 3005, Lab Unlimited UK, Camberley, UK) for 30 min at a rate of 3800 rpm. The centrifuged supernatants (layers of water) were filtered through a 0.2 µm membrane filter and injected onto the column.

#### 3.5.1. HPLC Analysis

The method was adapted to determine the concentration of linezolid in TPN mixtures (31). The analytical apparatus comprised an 1220 Infinity LC chromatography system (Agilent Technologies, Santa Clara, CA, USA) equipped with a DAD detector, a G1315C optical unit, an autosampler and a column oven. The mobile phase with a flow rate of 1 mL/min consisted of acetonitrile and potassium dihydrophosphate solution (50 mM) adjusted to pH 3.5 with 12% orthophosphoric acid (25:75 *V/V*). The stationary phase was octadecylsilyl silica gel for chromatography (Lichrospher^®^ 100 RP-18 column, 25 × 4 mm; 5 µm, Merck), at 25 ± 1 °C. The wavelength was 258 nm. The retention time for linezolid was 7.8 min and the analysis run time was 15 min. Each sample was injected in triplicate, and the volume of injection was 20 µL. The validation of the HPLC method concerned selectivity, precision, linearity, range and limits of detection and quantitation [49].

#### 3.5.2. Forced Degradation Study

Accurately weighed 4.8 mg of linezolid raw material was added to three covered tubes. Next 2.0 mL of 0.5 M sodium hydroxide, 0.5 M hydrochloric acid or 30% hydrogen peroxide was added and heated at 100 °C for 1 h. The solutions were cooled at room temperature, neutralized and diluted to 10.0 mL with the mobile phase. The degradation solutions were transferred into HPLC vials and analyzed according to the method described in Section 3.5.1.

**Figure 7:**
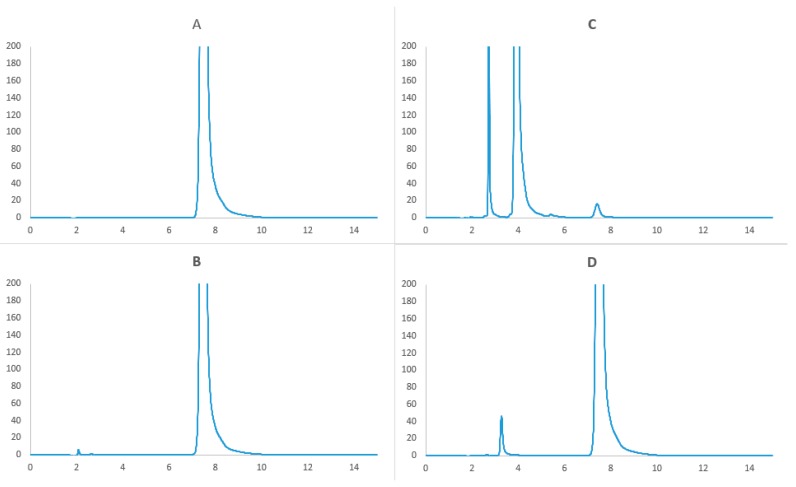
Chromatogram of (**A**) unstressed linezolid raw material (**B**) oxidation degradation of linezolid (**C**) basic hydrolysis of linezolid (**D**) acid hydrolysis of linezolid.

The HPLC study results presented in Figure 7 indicated that linezolid was unstable in stress conditions. The drug was hydrolyzed in acidic and basic media, and oxidized in the presence of 30% hydrogen peroxide. The chromatograms showed the peak of linezolid and those of degradation products, with lower retention times. These data indicated that the method adapted for the study was specific.

Linezolid is sensitive to hydrolysis. In acid environment we can observed one more peak on the chromatogram (Figure 7D). Linezolid is highly unstable in alkali condition. On the chromatogram we observed very small peak of linezolid and extra two peaks with lower retention time. Linezolid is also degraded in the presence of 30% hydrogen peroxide, but here was very little of degradation product, small peak with time retention about 2.1 min. These data also indicated specificity of the method that has been developed.

#### 3.5.3. Selectivity

To establish method selectivity the following samples were used: linezolid raw material, TPN mixture, TPN mixture with linezolid at t = 0 and t = 168 h, stored at 25 °C and protected from light, after separation of lipid emulsion.

**Figure 8:**
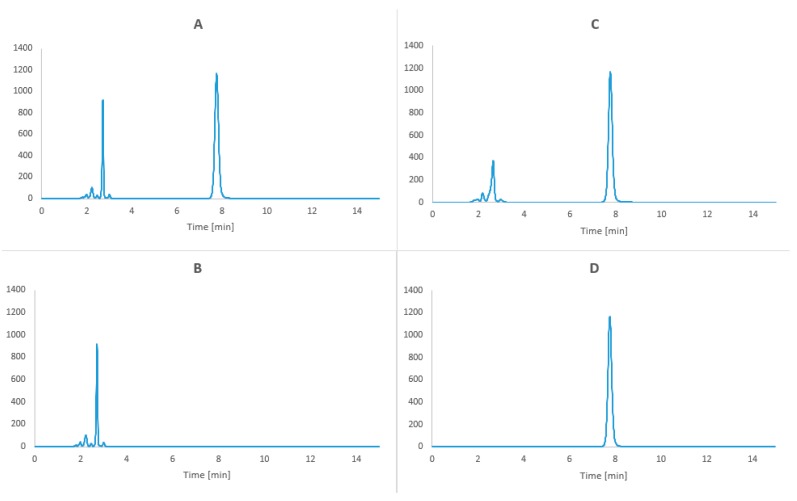
Chromatogram of (**A**) Linezolid in TPN mixture after extraction of lipid emulsion at t = 0 h (**B**) TPN mixture after extraction of lipid emulsion (t = 0) (**C**) Linezolid in TPN mixture at t = 168 h (25 °C with protection from light) after extraction of lipid emulsion (**D**) linezolid raw material.

Over a period of 15 min, the peaks of linezolid (retention time 7.8 min) and the amino acids (1.5–4.2 min) from the TPN mixture were observed on the chromatograms (Figure 8).

#### 3.5.4. Linearity

To evaluate linearity, measurements of five concentrations of linezolid were performed at a concentration from 25 to 125% of the nominal concentration of linezolid in TPN mixture. For each concentration three independent determinations were performed. For 100% nominal concentration of linezolid six determinations were conducted and used to establish method precision. The least squares method was used to calculate the regression parameters (y = ax + b, a ± Δa, and b ± Δb, standard errors Sa, Sb and Sy, and the correlation coefficient r; the a ± Δa, and b ± Δb were estimated for f = n − 2 degrees of freedom and α = 0.05) (Table 2). It was found that the value b is statistically insignificant; therefore the linearity of the method was described by the equation y = ax.

#### 3.5.5. Precision and Repeatability

Linezolid preparation (6.0 mL) was introduced into a 25 mL volumetric flask and diluted to 25.0 mL with TPN mixture. The so-obtained mixtures (3.0 mL) were introduced into 10 mL plastic vials and *n*-hexane (5.0 mL) was added. The remaining procedure followed that designed for linearity. The intra-day repeatability of the method was assessed by extracting and measuring six samples of the same concentrations during a single analytical run. The inter-day repeatability was investigated by analyzing six aliquots of the same samples on different days. Next day a repeatability of the method was established (Table 2).

#### 3.5.6. Detection and Quantitation Limits

Detection and quantitation limits were calculated from the following formulas DL = 3.3 sy/a and QL = 10 sy/a, respectively (sy—standard deviation, a—calibration curve slope) (Table 2).

#### 3.5.7. Range

The range of an analytical procedure is the interval between the upper and lower concentrations of the analyte in the sample (including these concentrations) for which it was demonstrated that the analytical procedure had a suitable level of precision, accuracy and linearity.

In the HPLC method for the determination of linezolid stability in TPN mixture, the method range was from 0.04 to 0.72 mg/mL.

A summary of validation parameters for the HPLC method used for linezolid determination in TPN mixtures is presented in Table 2.

#### 3.5.8. pH Measurement

The pH was measured in triplicate for each mixture with a Seven Compact pH/ion S220^®^ meter (Mettler Toledo, Columbus, OH, USA). A pH measurement was carried out each day of storage of the TPN mixtures with and without linezolid. Before pH measurement, a three-point calibration of the pH-meter was performed, with a buffer solution of pH 10.00, 7.00 and 4.00. Each sample was measured in triplicate.

#### 3.5.9. Particle Size Measurements

Particle size measurements were based on a dynamic light scattering method using Zetasizer Nano ZS apparatus (Malvern Instruments Ltd., Malvern, UK) with the particle size option. The size corresponded to the hydrodynamic diameter of the particle in the emulsion. The samples were diluted at a ratio of 1:10 (*V/V*) with water for injection. The aliquot was then placed in a low-volume polypropylene cuvette, checked for the absence of air bubbles, and placed in the Zetasizer sample holder for measurement from 4 to 90 °C with equilibration times of 120 s at each temperature. Five measurements were made for each sample. Each measurement included 50 runs of 10 s, at intervals of 30 s. All the measurements were performed in triplicate and the results were expressed in terms of the average plus standard deviation values

#### 3.5.10. Zeta Potential Measurements

The surface electric properties of lipid emulsion particles were determined using a Laser Doppler-Electrophoresis (LDE) method. Electrically charged particles of the lipid emulsion migrate towards the electrode in response to the electric field applied and their velocity can be measured and expressed as their mobility. The direction and velocity of the motion are a function of the particle charge, the medium, and the electric field strength. Zeta potential is proportional to the electric potential of the particle in the slipping plane. The particle velocity was measured by observing the Doppler shift in the scattered light using Malvern Zetasizer Nano ZS apparatus (Malvern Instruments Ltd.) at 25 ± 1 °C, and the zeta potential of the particles was automatically calculated using the Smoluchowsky formula and expressed in mV. Zeta potential evaluation was carried out for parenteral nutrition formulations with and without linezolid, immediately after preparation and each day of storage. Samples were diluted 1:10 (*V/V*) with water for injection. DTS1070 disposable folded capillary cells were used to perform LDE measurements in an auto mode analysis model with automatic measurement duration.

## Figures and Tables

**Table 1:** Composition of Total Parenteral Nutrition mixtures.

TPN Mixture Elements	Composition					
	Basic	Immuno-modulatory	High-Electrolyte	Low-Electrolyte	High-Energetic	Low-Energetic
Aminoplasmal^®^ B. Braun 10% E	600	600	600	600	600	0
Aminoplasmal HEPA^®^ 10%	0	0	0	0	0	600
Glucose 40%	481.49	481.49	481.49	481.49	581.49	431.49
LIPIDem^®^	0	300	0	0	0	0
Lipofundin^®^ MCT/LCT 20%	300	0	300	300	350	150
Aqua pro iniectione	372.77	372.77	308.77	405.77	222.77	572.77
Natrium chloratum 10%	57.74	57.74	77.74	37.74	57.74	57.74
Kalium chloratum 15% WZF	40	40	60	35	40	40
Calcium gluconate 10%	20	20	30	20	20	20
Glycophos	20	20	30	15	20	20
Inj. Magnesii sulfurici 20%	8	8	12	5	8	8
Linezolid Polpharma	600	600	600	600	600	600
Total volume [mL]	2500	2500	2500	2500	2500	2500

**Table 2:** A summary of validation parameters for the HPLC method used for linezolid determination in TPN mixtures.

Parameters	Acceptance Criteria	Results
Selectivity Influence of interfering substances	Separation of linezolid peak from peaks of TPN mixture ingredients	Acceptable Amino acids, t_R_ 1.5–4.2 min Linezolid t_R_ = 7.8 min
Linearity Correlation coefficient r for equation y = ax	r ≥ 0.990	Acceptable y = (29252.4 ± 286.5)x; r = 0.9998
Repeatability - intra-day - inter-day	RSD ≤ 5%	Acceptable RSD = 0.31% RSD = 0.23%
Limits of detection and quantitation	QL = 0.013; DL = 0.040	

## Supplementary Materials

The following are available online. Scheme S1. Diagram of study design.
