# Supplementary material for: Physicochemical Compatibility and Stability of Linezolid with Parenteral Nutrition
Source: Molecules. 2019 Mar 29;24(7):1242. doi: 10.3390/molecules24071242 (PMC6480230; doi:10.3390/molecules24071242)
Supplement: Supplementary file 1 [file molecules-24-01242-s001.pdf]

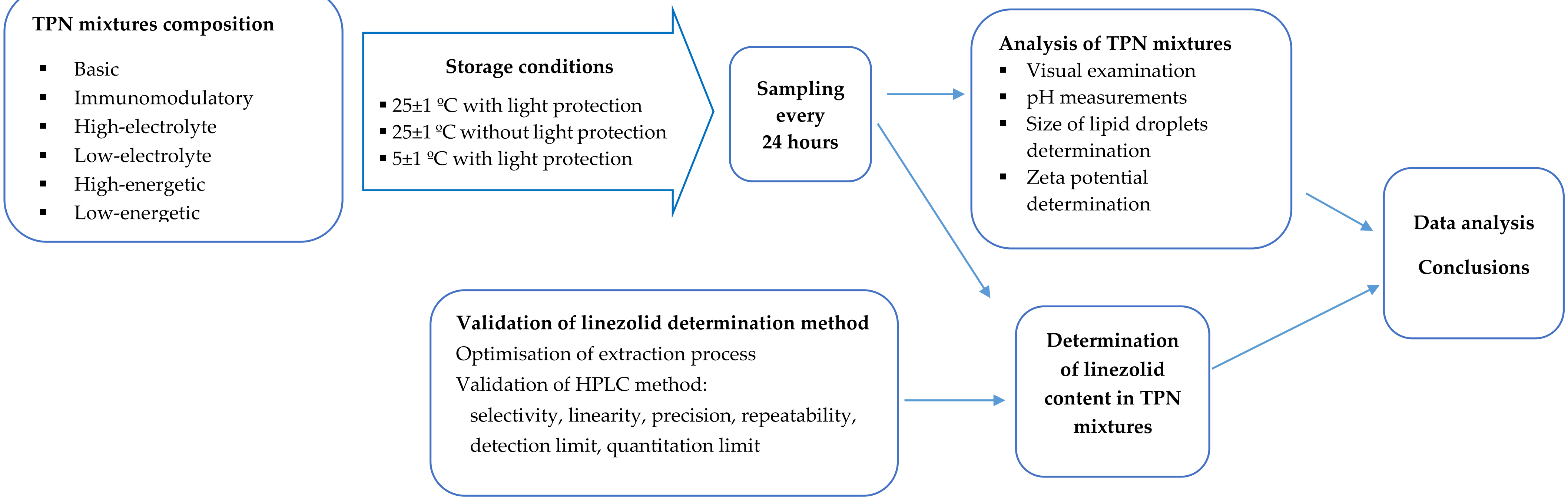

Scheme 1. Diagram of study design (TPN – total parenteral nutrition; HPLC – high-performance liquid chromatography)
